# Kojic acid-mediated damage responses induce mycelial regeneration in the basidiomycete *Hypsizygus marmoreus*

**DOI:** 10.1371/journal.pone.0187351

**Published:** 2017-11-08

**Authors:** Jinjing Zhang, Hui Chen, Mingjie Chen, Hong Wang, Qian Wang, Xiaoxia Song, Haibo Hao, Zhiyong Feng

**Affiliations:** 1 National Research Center for Edible Fungi Biotechnology and Engineering, Key Laboratory of Applied Mycological Resources and Utilization, Ministry of Agriculture and Shanghai Key Laboratory of Agricultural Genetics and Breeding, Institute of Edible Fungi, Shanghai Academy of Agricultural Sciences, FengXian District, Shanghai, China; 2 Microbial Resources and Application Laboratory, School of Food Science and Engineering, Hefei University of Technology, Hefei, China; 3 College of Life Science, Nanjing Agricultural University, XuanWu District, Nanjing, China; Oregon State University, UNITED STATES

## Abstract

Mechanical damage can induce fruiting body production in fungi. In this study, the antioxidant kojic acid (KA) was found to enhance injured mycelial regeneration and increase fruiting body production in *Hypsizygus marmoreus*. KA reduced the level of reactive oxygen species (ROS), which are harmful to mycelia when excessively generated by mechanical damage. Moreover, KA increased catalase and superoxide dismutase activities and glutathione and ascorbic acid contents by up-regulating antioxidant gene expression. These results suggest that KA promotes mycelial regeneration in response to damage by activating a “stress signal” and enhances the ability of *H*. *marmoreus* to resist oxidative damage by invoking the antioxidant system. In addition, KA increased the content of extracellular ATP, which serves as a “stress signal” in response to injury, and modulated ROS signaling, decreasing NADPH oxidase gene expression and ROS levels in the mycelial-regeneration stage. KA treatment also up-regulated the MAPK, Ca^2+^ and oxylipin pathways, suggesting their involvement in the damage response. Furthermore, laccase and cellulase activities were stimulated by KA at different developmental stages. These results demonstrate that KA regulates gene expression and activates pathways for mycelial wound healing, regeneration of damaged mycelia and reproductive structure formation in the basidiomycete *H*. *marmoreus*.

## Introduction

Fruiting body production is induced in maturing mycelia upon exposure to adverse environmental conditions, such as mechanical damage, which affect fungal growth, development, and reproduction [1]. Although it is known that mechanical damage results in fruiting body production in the damaged area in *Schizophyllum commune* [2], knowledge of how other basidiomycetes respond to injury is limited. In ascomycetes, mechanical damage induces sclerotia formation [3], and Fester and Hause [4] found that mechanical damage induces reactive oxygen species (ROS) accumulation in the mycorrhizal fungus *Glomus intraradices*. In addition, the mechanism by which injury induces the formation of conidia in the damaged area was studied recently in *Trichoderma atroviride* [5–7], and Hernández-Oñate et al. [6, 8] suggested that signaling molecules and pathways involved in the injury response of this fungus, including injury, healing, and regeneration signals, share common features with injury responses in plants and animals.

Indeed, animals and plants have developed mechanisms to rapidly and reliably respond to mechanical damage [8]. Similarly, in fungi, extracellular ATP (eATP) serves as an “injury signal” that triggers asexual development as an injury response [7]. ROS production also plays an important role in inducing healing and regeneration in the damaged area [9–10], and there are several important signaling pathways involved in the injury response, including ROS, MAPK, and calcium signaling [7]. However, over-production of ROS also affects cellular components through the oxidation of proteins, DNA, lipids, and other biomolecules, which might induce cell damage or death [11]. To achieve normal growth, organisms have developed mechanisms for regulating ROS levels [12]. For example, fungi can regulate their development by maintaining ROS at a proper level [6, 13], with important roles for antioxidant enzymes such as catalase (CAT) and superoxide dismutase (SOD) in scavenging these harmful molecules [14–15].

Kojic acid [5-hydroxy-2-(hydroxymethy1)-y-pyrone] (KA), a fungal secondary metabolite, is produced by many species of *Aspergillus* and *Penicillium* [16]. KA is now used in a variety of applications, including as an antibiotic [17], an additive to prevent the browning of food materials, and an antioxidant [18]. Recent studies have focused on its antibiotic activity, which may help an organism resist oxidative stress via ROS scavenging [19]. In fungi, KA was shown to increase cellular resistance to oxidative stress and to reduce damage [20]. In *Aspergillus parasiticus* and *Aspergillus flavus*, increases in KA levels regulate mycelial growth and conidial development by mediating the oxidative state [21]. KA can also induce repair of damaged tissues [22].

In *Hypsizygus marmoreus*, fruiting body initiation in sawdust medium requires mechanical damage induced by scraping mature mycelia, though the mechanism regulating this injury response remains unknown. In this study, we found that adding the antioxidant KA to scraping-damaged *H*. *marmoreus* mycelia induced mycelial healing and regeneration and stimulated primordium initiation. Furthermore, KA regulated “injury signals”, such as eATP and ROS, and increased antioxidant activity. KA also up-regulated expression of genes involved in certain signaling pathways associated with the mechanical damage response.

## Materials and methods

### Strain and culture conditions

*H*. *marmoreus* samples were obtained from the China General Microbiological Culture Collection Center (Beijing) (no. CGMCC5.01974). After culturing on PDA (potato dextrose agar; BD company, USA) medium for two weeks, the strain was transferred to PDB (PD broth; BD company, USA) and cultured for another week. The liquid culture was inoculated onto sawdust medium (sterilized at 121°C and 0.1 MPa for 2.5 h) in a 1100-mL bottle and cultured in the dark at 25°C. After growth for 80 d, the surface mycelia were mechanically damaged using a scraping machine, and the damaged mycelia were supplied with 15 mL tap water (CK) or 10 mM KA. The bottles were placed under reproduction conditions reported previously [23]. On the 22nd day, when the fruiting bodies had matured, the production and number of fruiting bodies were calculated.

### KA detection assays

Samples at the mycelial regeneration stage (H-M), pigmentation stage (H-V), and primordium stage (H-P) were collected from the CK and KA groups, and extracellular KA concentrations were assessed. Mycelia cultured on sawdust medium for 20, 60 and 80 d were also collected for extracellular KA concentration determination. KA levels were evaluated using the colorimetric method [16]. KA forms a chelated compound with ferric ions, subsequently generating a red color, as measured at 495 nm. The substrates from different developmental stages were collected, 25 g of which was prepared in 50 mL of ddH_2_O in 250-mL flasks. The mixture was shaken at 200 rpm for 30 min at 25°C followed by filtration and centrifugation at 6000×g for 20 min. A 1-mL aliquot of the liquid supernatant was mixed with 4 mL of ddH_2_O. The reaction for KA detection contained 2 mL diluted liquid supernatant, 3 mL 1% (w/v) aqueous FeCl_3_ and 5 mL ddH_2_O.

### RNA-Seq sample preparation, sequencing and analysis

After scraping mycelia, the damaged mycelia were treated with 15 mL 10 mM KA or 15 mL tap water as a control. After approximately 2 days of regeneration (H-M), regenerated mycelia were collected with the sawdust medium. On approximately the 6th day, the white mycelium turned brown (H-V); the primordium appeared on approximately the 10th day (H-P). The brown mycelia with little sawdust medium and primordia were then collected. To obtain an overview of the effects of KA on gene expression profiles of *H*. *marmoreus* during the three developmental stages, cDNA samples were prepared from the H-M, H-V and H-P stages of the CK and KA groups and frozen at -80°C for RNA extraction.

Total RNA was extracted with TRIzol (Takara), reverse-transcribed to cDNA, and sequenced as described previously [23]. Two independent repetitions for each sample were sequenced separately using the Illumina HiSeqTM 2000 sequencing platform. All data were analyzed as described previously [23]. Reads of 2×100 bp were obtained, and clean reads were used to perform RNA *de novo* assembly with Trinity [24]. Genes were predicted and annotated using local BLASTX programs against the National Center for Biotechnology Information (NCBI) nr/nt, SwissProt, and STRING databases [25]. Differentially expressed genes (DEGs) between the two samples were analyzed using RSEM [26] and edgeR [27], and the expression level of each unigene was assessed according to fragments per kilobase of exon per million reads mapped (FPKM) [28]. The raw data for the three developmental stages of the CK and KA treatments have been submitted separately to NCBI under the accession number SRA454268 and the bioproject number PRJNA339890.

### Antioxidant enzyme detection assays

To examine changes in antioxidant enzyme activities, mycelia of the CK and KA groups were collected at the three developmental stages (H-M, H-V and H-P); 1.0 g was ground in liquid nitrogen, 9.0 mL normal saline was added, and the sample was centrifuged at 2500×g for 10 min. The supernatant (50 μL) was used to detect CAT and SOD activities. CAT activity was detected according to the instructions of the CAT assay kit (Visible light) (Nanjing Jiancheng Bioengineering Institute, Nanjing, China). One unit of CAT was defined as the amount of enzyme that decomposed 1 μmol of H_2_O_2_, as monitored at 405 nm. SOD activity was detected as described by the Total SOD assay kit (hydroxylamine method) (Nanjing Jiancheng Bioengineering Institute, Nanjing, China). One unit of SOD activity was defined as the amount of SOD that inhibited 50% of hydroxylamine oxidation per gram of tissue in 1 mL of solution, as monitored at 550 nm.

### Laccase and cellulase activity detection

Laccase activity was detected at the three developmental stages (H-M, H-V and H-P) in samples treated or not with KA. Samples collected from the three developmental stages were pretreated using a method developed for *Pleurotus ostreatus* [29]. After treatment, the filtrate was recovered and centrifuged at 4°C at 6000×g for 20 min. laccase activity was detected according to a previously described method [30]. The change in absorbance at 420 nm was recorded for 3 min with ABTS (2, 2'-azino-bis(3-ethylbenzothiazoline-6-sulphonic acid)) as the substrate. One unit activity was defined as the amount of enzyme that oxidized 1 μmol of ABTS/min.

The same samples were also used to detect extracellular cellulase activity by the 3, 5-dinitrosalicylic acid method, which assesses the reducing sugar concentration as a result of by cellulase catalysis. The liquid supernatant was used for detecting cellulase activity, as described by the cellulase assay kit manual (Nanjing Jiancheng Bioengineering Institute, Nanjing, China) and monitored at 550 nm. One unit of cellulase activity was defined as the amount of gram of mycelial protein that produced 1 μg of glucose per minute.

### ROS detection assays

To evaluate changes in ROS, injured mycelia of the CK and KA groups were collected at 15, 30, and 60 min after damage and immediately frozen at -80°C. The samples were ground with liquid nitrogen. Inhibition of superoxide anion (O^2-^) activity was performed as described by the Inhibition and produced O^2-^ assay kit (Nanjing Jiancheng Bioengineering Institute, Nanjing, China). In this reaction system, the amount of O^2-^ inhibited by 1 g of mycelial protein at 37°C for 40 min was related to the amount of O^2-^ inhibited by 1 mg of Vc, as monitored at 550 nm, and this value was consider as one unit of O_2_^-^-inhibiting activity. Hydrogen peroxide (H_2_O_2_) detection was performed as described by the H_2_O_2_ assay kit (Nanjing Jiancheng Bioengineering Institute, Nanjing, China), as monitored at 405 nm; hydroxy free radical (-OH) detection was performed as described by the -OH assay kit (Nanjing Jiancheng Bioengineering Institute, Nanjing, China), as monitored at 550 nm. The O_2_^-^-inhibiting activity and H_2_O_2_ and -OH concentrations were calculated using the mycelial protein concentration.

### Quantitation of reduced glutathione and ascorbic acid

Mycelia of the CK and KA groups the three stages (H-M, H-V and H-P) of collected at the three developmental stages (H-M, H-V and H-P) were also to detect reduced glutathione (GSH) and ascorbic acid (AsA). The mycelia were ground in liquid nitrogen using a mortar and pestle, suspended in 6% (w/v) m-phosphoric acid containing 1 mM ethylenediaminetetraacetic acid (EDTA) and centrifuged at 12,000×g for 10 min. Total GSH was detected using a described method [31]. GSH contains sulfhydryls (–SH), which oxidize DTNB (5’, 5’-dithiobis-20-nitrobenzoic acid) to NTB (50-dithiobis-20-nitrobenoic acid), generating a yellow color in an aqueous solution. The GSH level was detected at 412 nm using a spectrophotometer.

In addition, crushed CK and KA mycelia were homogenized in 5% (w/v) m-phosphoric acid on ice. The homogenate was centrifuged at 12,000×g for 15 min. Total AsA was determined by a described method [32] based on the reduction of Fe^3+^ to Fe^2+^ with ascorbic acid in acid solution followed by the formation of a red chelate between Fe^2+^ and 2,20-bipyridyl. Total AsA was determined in a reaction mixture consisting of 0.2 mL supernatant, 0.5 mL 150 mM potassium phosphate buffer (pH 7.4) containing 5 mM EDTA, and 0.1 mL 10 mM dithiothreitol (DTT) to reduce DHA to AsA. AsA levels were detected at 525 nm using a spectrophotometer.

### Extracellular ATP (eATP) detection assays

To assess eATP changes, CK and KA injured mycelia with sawdust medium were collected at 15, 30, and 60 min after damage, and the samples were weighed to exactly 1.0 g. The 1.0-g substrate mixtures were added to 9.0 mL boiling water in a 50-mL centrifuge tube and boiled for 10 min. The mixture was then filtered, and the filtered solution was centrifuged at 3000×g for 10 min. The liquid supernatant was used for eATP content detection, as described in the ATP assay kit (Nanjing Jiancheng Bioengineering Institute, Nanjing, China) and monitored at 636 nm.

### Total protein detection

The mycelial protein concentration used for calculations was detected at 595 nm as described by the Total protein quantitative assay kit (Coomassie Brilliant Blue) (Nanjing Jiancheng Bioengineering Institute, Nanjing, China). The standard protein concentration used in this assay kit was 0.563 g/L, and Coomassie brilliant blue was employed.

### Quantitative reverse transcriptase polymerase chain reaction (qRT-PCR) validation

Approximately 2 μg total RNA from the CK and KA groups at the three stages (H-M, H-V and H-P) was reverse-transcribed by M-MLV reverse transcriptase (Takara) using oligo (dT) as the primer. Genes of interest were subjected to qRT-PCR analysis. The primers and accession numbers of these genes and the internal reference gene (18S ribosomal RNA) are listed in S1 Table. Experiments were carried out using a Realplex2 System (Eppendorf, Germany), and the amplification conditions were as follows [23]: 95°C for 10 min, 40 cycles of 95°C for 30 s, 55°C for 30 s, and 72°C for 30 s. Three independent biological replicates were performed for each gene. Relative gene expression was analyzed using the 2^-ΔΔCt^ method [33].

### Data presentation and statistical analysis

Values are shown as the mean ± SD of three independent experiments with three replicates each. Differences among treatments were analyzed by one-way analysis of variance (ANOVA) combined with Duncan’s multiple range test at a probability of P < 0.05.

## Results

### Effects of KA on mycelial regeneration and fruiting body production in *H*. *marmoreus*

**Fig 1:**
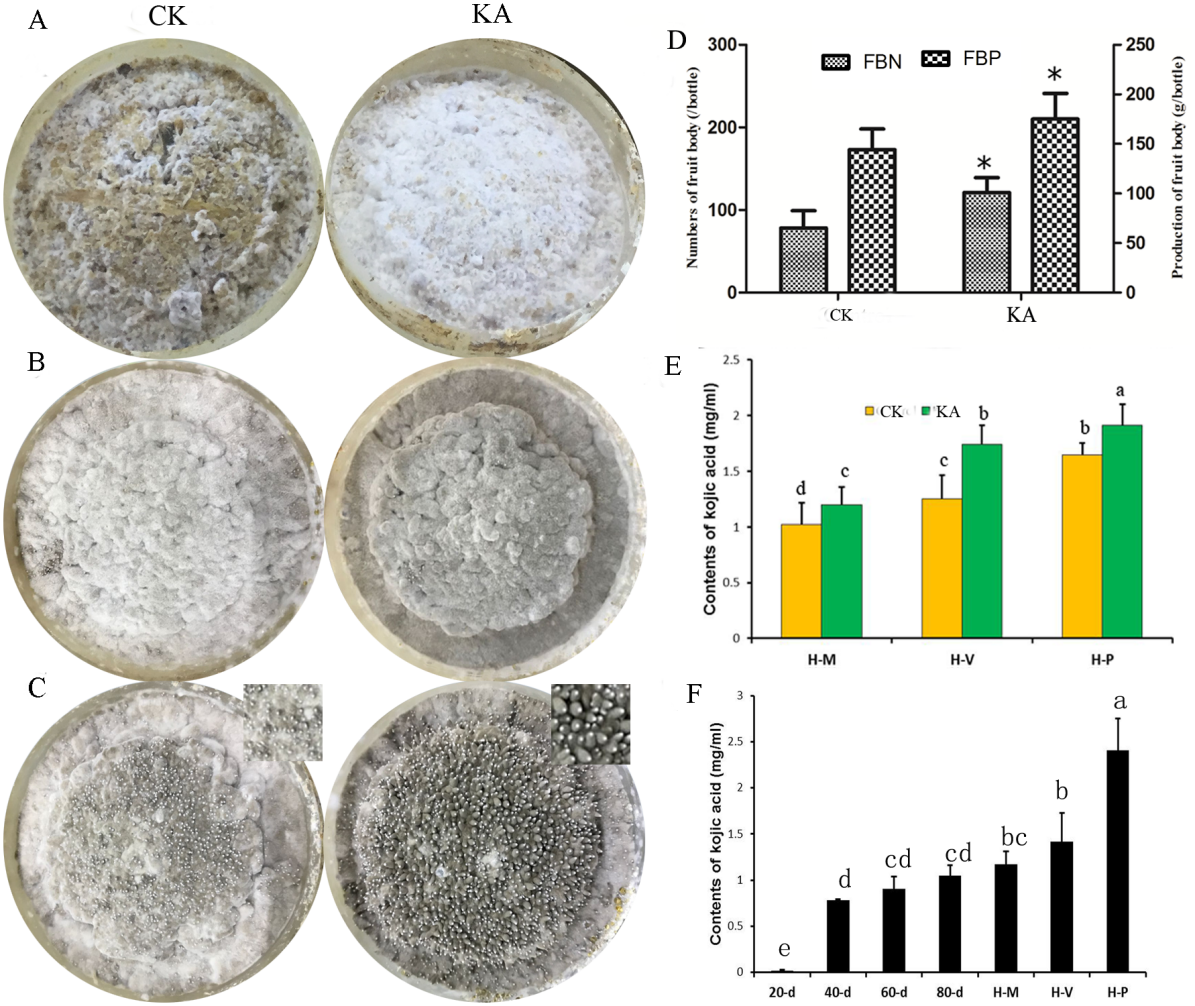
Effects of kojic acid on fruiting body development in *H*. *marmoreus*. A: Mycelial regeneration (H-M); B: mycelial pigmentation (H-V); C: primordium (H-P); D: the production and number of fruiting bodies; E: the content of kojic acid (KA) at different developmental stages; F: the content of KA during the developmental stages of *H*. *marmoreus*. CK: control group; FBN: fruiting body number, FBP: fruiting body production, 20–80 d: the time for mycelium growth in sawdust medium. The data are presented as the means ± SD of three independent experiments. Bars with different letters or asterisk denote a statistically significant difference compared with the control according to multiple comparisons (P < 0.05).

Before the reproductive growth of *H*. *marmoreus*, external mycelia were machine scraped, and 15 mL of CK or 10 mM KA was added. After the mechanical damage stimulus, the mycelia were transferred to a reproductive culture room. In this study, we found that adding 10 mM KA induces damaged mycelia to heal and regenerate quickly (Fig 1A), and mycelial pigmentation and primordium formation were also stimulated when compared to CK, which was only treated with tap water (Fig 1B and 1C). Brown mycelia in the CK group were observed when the mycelia in the KA group were completely dark brown (Fig 1B). When primordia were approximately 0.5 cm long in the KA group, those in the CK group were barely visible. Moreover, fruiting bodies matured much earlier in the KA group than in the CK group (Fig 1C).

KA treatment also significantly increased the production and number of fruiting bodies in *H*. *marmoreus* (Fig 1D). However, no significant differences were found when KA was added at any other developmental stage (data not shown).

### KA levels in the developmental stages of *H*. *marmoreus*

KA is a major secondary metabolite produced by fungi [16]. We detected KA in the substrate medium of *H*. *marmoreus* and found significant differences in the concentration, along with mycelial vegetative growth and fruiting body development (Fig 1F). In addition, KA production was significantly increased at the three developmental stages (H-M, H-V and H-P) upon KA addition (Fig 1E). These results were in accordance with reports showing that KA production is triggered by KA supplementation [34–35].

### KA alters the transcriptome of *H*. *marmoreus*

**Fig 2:**
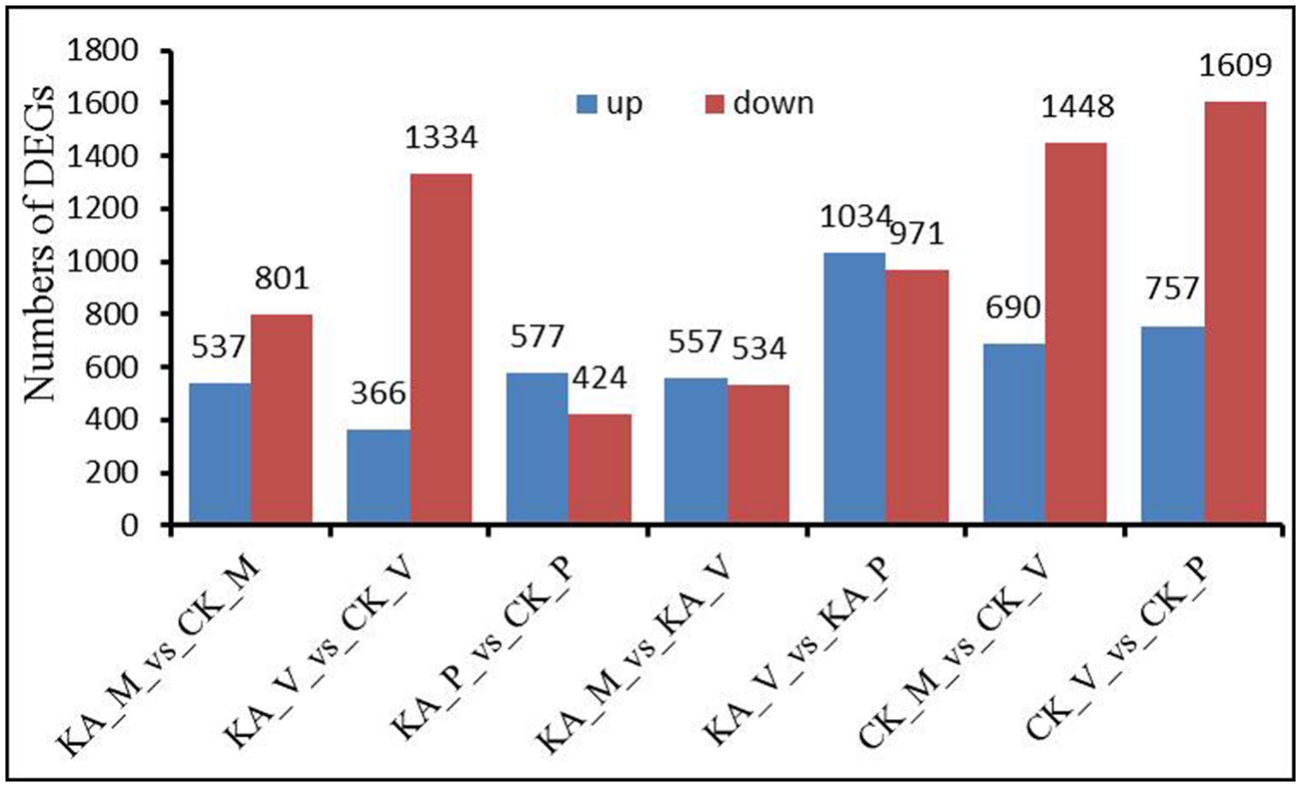
Number of differentially expressed unigenes in each comparison. KA_M_vs_CK_M: the number of up- and down-expressed unigenes in the comparison KA_M to CK_M; KA_V_vs_CK_V: the number of up- and down-expressed unigenes in the comparison KA_V to CK_V; KA_P_vs_CK_P: the number of up- and down-expressed unigenes in the comparison KA_P to CK_P; KA_M_vs_KA_V: the number of up- and down-expressed unigenes in the comparison KA_M to KA_V; KA_P_vs_KA_V: the number of up- and down-expressed unigenes in the comparison KA_P to KA_V; CK_M_vs_CK_V: the number of up- and down-expressed unigenes in the comparison CK_M to CK_V; CK_V_vs_CK_P: the number of up- and down-expressed unigenes in the comparison CK_V to CK_P.

To detect the role of KA in mycelial regeneration and fruiting body production in *H*. *marmoreus*, transcript profiling was performed at the stages of mycelial regeneration (H-M), mycelial pigmentation (H-V) and primordium (H-P) in the CK and KA groups. In total, 24,529 unigenes were detected, as listed in S1 Dataset. Gene Ontology (GO) assignments were used to classify the functions of the annotated unigenes related to other fungi. Among the annotated unigenes, 20,659 were categorized into 41 functional groups (S1 Fig). Moreover, 5246 identified unigenes were assigned to 24 functional classes in clusters of orthologous groups of proteins (COG) analysis (S2 Fig). KA altered the number of DEGs and the ratio of up-regulated to down-regulated unigenes (Fig 2). Moreover, during the transitions from H-M to H-V and from H-V to H-P, the number of up-regulated unigenes in KA treatment exceeded that of down-regulated unigenes. In the CK group, fewer up-regulated unigenes than down-regulated unigenes were observed in both transition phases. Although the numbers of up-regulated unigenes were fewer in the KA-M transition compared with the CK-M transition and in the KA-V transition compared with the CK-V transition, there were more up-regulated unigenes in the KA-P transition than in the CK-P transition. Our findings suggest a predominantly positive regulatory role for KA in *H*. *marmoreus* primordium initiation.

**Fig 3:**
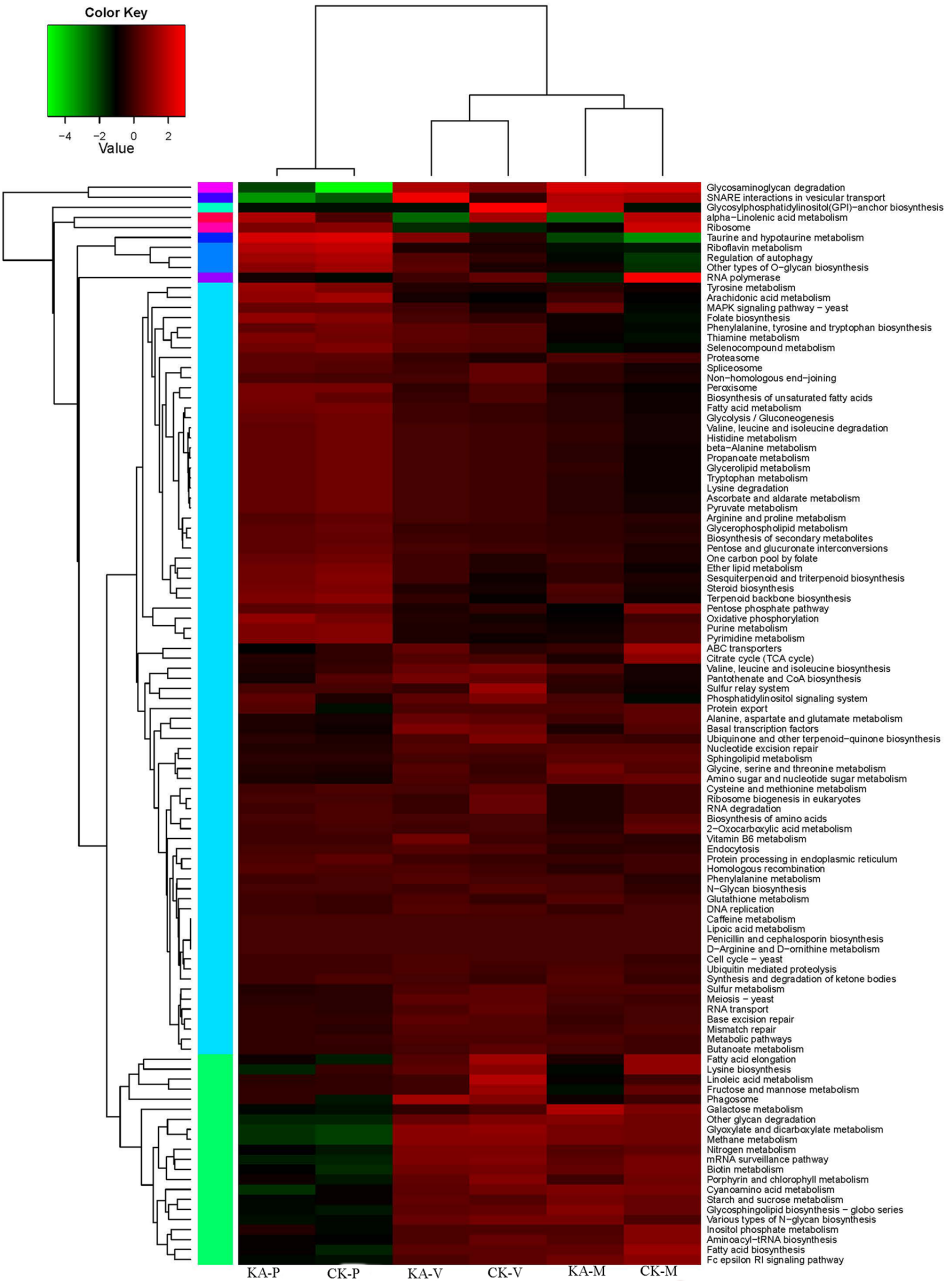
KEGG annotation of differentially expressed genes (DEGs). A heatmap showing the annotated pathways of DEGs in the six samples of *H*. *marmoreus*. Different colors represent different expression levels. Green represents down-regulated expression and red up-regulated expression. Each row represents a different pathway. The heatmap was constructed based on the log10 values of the FPKM of all DEGs related to this pathway in the H-M, H-V and H-P developmental stages.

We analyzed the metabolic and regulatory pathways related to the identified DEGs, and heatmap analysis was performed based on the total FPKM values of all the DEGs in each pathway (Fig 3 and S2 Dataset). Most of these pathways, including the MAPK signaling pathway, glycerolipid metabolism, glycerophospholipid metabolism, peroxisomes, ascorbate and aldarate metabolism, pyruvate metabolism, glutathione metabolism, nitrogen metabolism, starch and sucrose metabolism, and the cell cycle, were up-regulated in the KA group. In contrast, pathways of fatty acid elongation, lysine biosynthesis, fatty acid biosynthesis, and protein export were up-regulated in the CK group.

**Fig 4:**
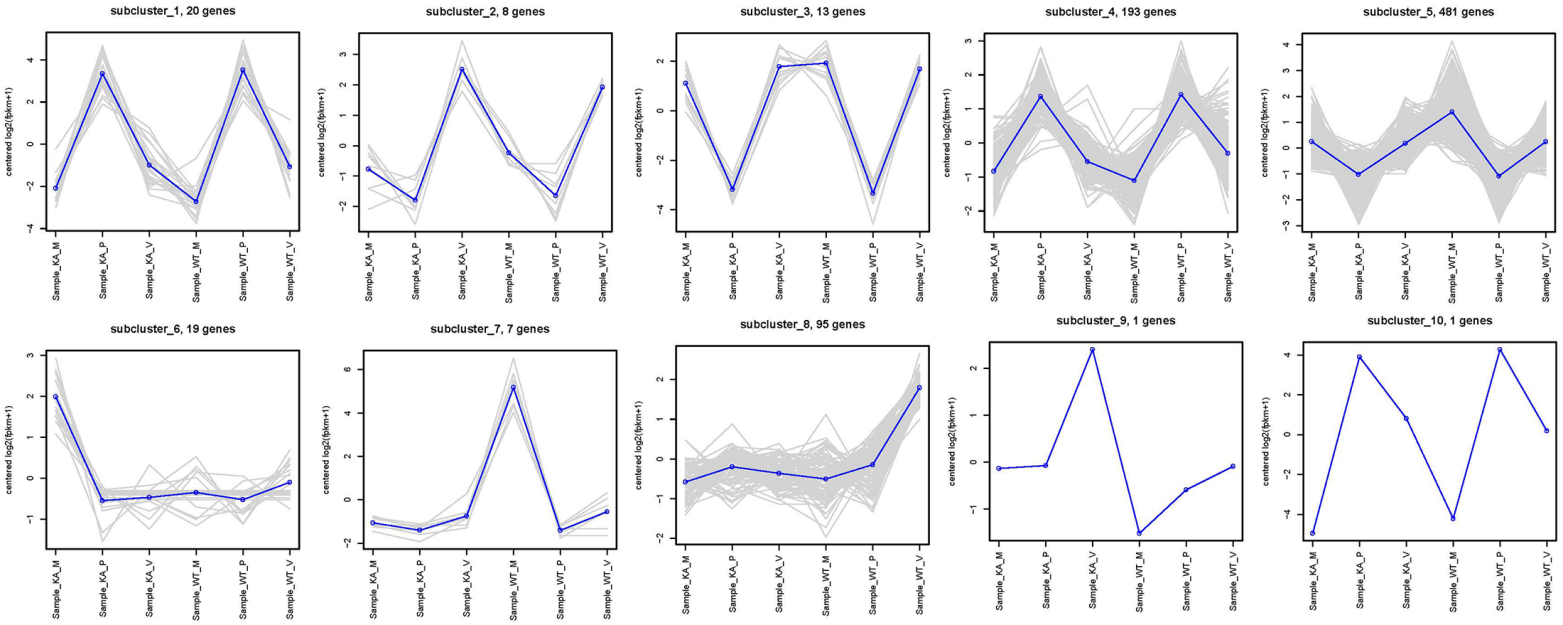
Cluster analysis of the gene expression profiles in the three stages after kojic acid treatment. Cluster analysis was performed on 838 genes detected in the mycelial regeneration, mycelial pigmentation, and primordium stages. The log2 value of the FPKM for each gene was used for the hierarchical clustering analysis. These genes were classified into 10 regulatory patterns.

The DEGs were assigned to 10 groups according to their transcript levels in the CK and KA samples, and their kinetics over the three stages were analyzed (Fig 4 and S3 Dataset). The majority of genes were classified into subclusters 4 and 5, with opposite transcript patterns. In subclusters 1, 2, 3, 4, 5, and 10, the pattern of the transcript levels in the three developmental stages was consistent. However, in subcluster 7, the transcript levels of genes at H-M in the CK group were higher than those in the KA group, including DNA-binding protein HU, ribosomal subunit interface protein, and DNA-directed RNA polymerase subunit alpha. In subcluster 6, the transcript levels of genes at H-M in the KA group were higher than those in the CK group, including laccase, NAD dependent epimerase/dehydratase family protein, oxidoreductase, short chain dehydrogenase/reductase family, and hydrophobin. In subcluster 8, KA altered gene transcript patterns, with the highest transcript level at H-V in the KA group and at H-P in the CK group.

**Fig 5:**
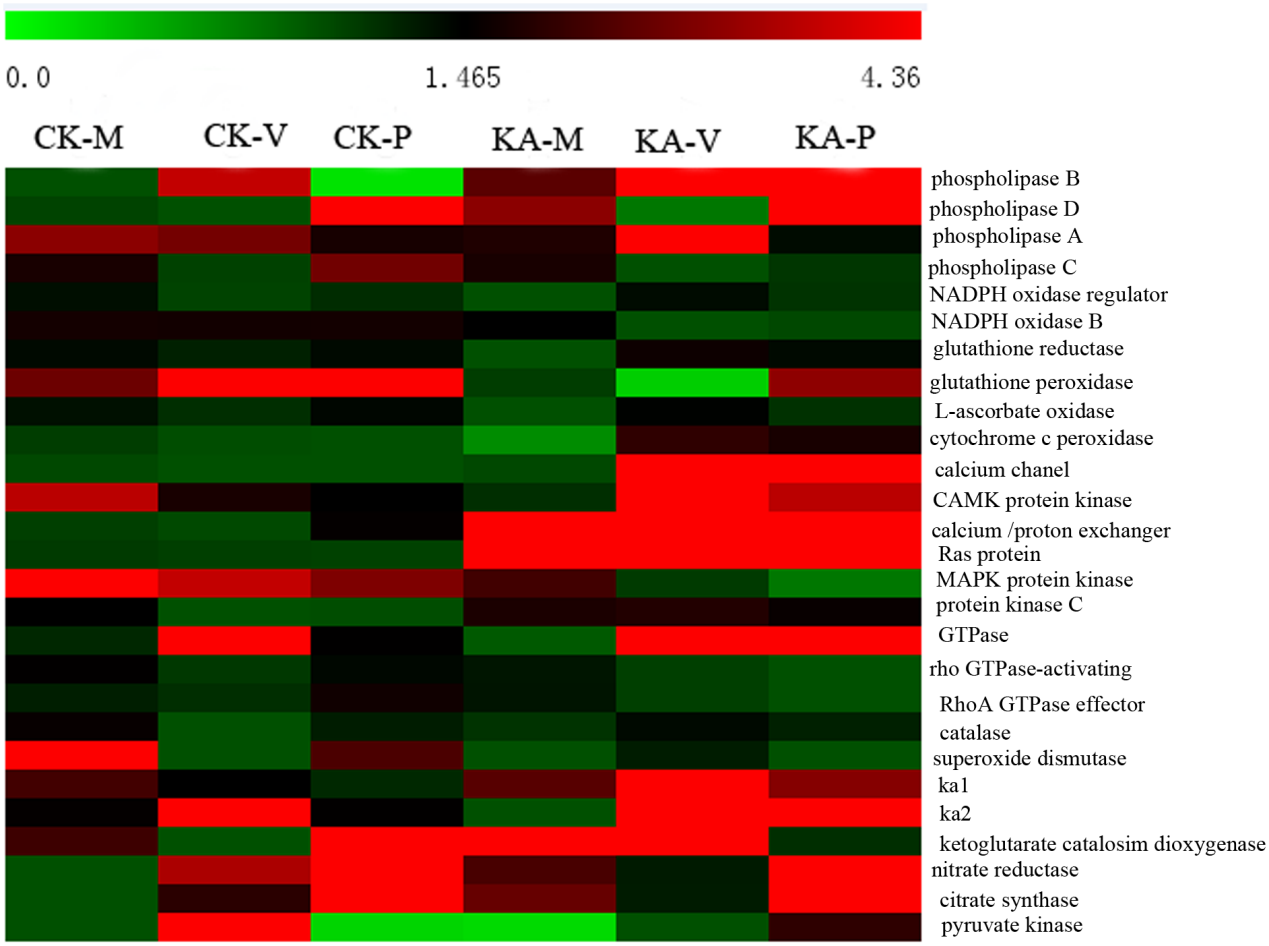
DEGs between four developmental stages in *H*. *marmoreus*. Each column represents an experimental sample (*e*.*g*., CK-M, CK-V, CK-P, KA-M, KA-V and KA-P) and each row a gene. Expression differences are shown in different colors. Red indicates high expression and green low expression.

The effects of KA on gene expression at different developmental stages were analyzed by co-expression analysis [23]. Heatmap analysis was performed based on the FPKM values of 27 DEGs involved in signal transduction, energy metabolism, cell cycle, and secondary metabolism (Fig 5). The functional annotations for these unigenes are listed in S2 Table. As shown in Fig 5, genes involved in carbon metabolism, such as unigenes encoding pyruvate kinase and glyceraldehyde 3-phosphate dehydrogenase, were up-regulated in KA-M compared with CK-M. Genes involved in signal transduction, encoding NADPH oxidase regulator NoxR, CAMK/CAMKL/GIN4 protein kinase, CMGC/MAPK protein kinase, and alpha-ketoglutarate catabolism dioxygenase, were up-regulated in the KA groups compared with the CK groups, and genes encoding antioxidant enzymes, including catalase and superoxide dismutase, were also up-regulated by KA treatment. In accordance with the gene expression results, the activities of CAT and SOD were significantly higher in the KA group than in the CK group at the three developmental stages, except that CAT activity showed no significant change at the H-P stage (Table 1). The contents of the antioxidants GSH and AsA also increased significantly due to KA treatment (Table 1). In addition, genes involved in KA metabolism such as Zn(2)-Cys(6) binuclear cluster domain-containing protein (KA1) and C_2_H_2_-type Zn-finger protein (KA2) were up-regulated by KA.

**Table 1:** The effects of kojic acid on antioxidative enzyme activity and antioxidant contents at three stages in *H*. *marmoreus*.

Stages	SOD activity (U/mgprot)		CAT activity (U/mgprot)		GSH contents (μmol/g)		AsA contents (μmol/g)	
	Control	KA	Control	KA	Control	KA	Control	KA
**H-M**	203.07±10.2^c^	328.1±8.0^a^	900.5±19.1^b^	1161.3±16.7^a^	0.168±0.001^f^	0.288±0.012^d^	0.757±0.02^e^	2.457±0.10^c^
**H-V**	198.3±13.8^c^	235.8±4.8^b^	316.4±13.9^d^	492.2±14.0^c^	0.213±0.003^e^	0.432±0.003^b^	0.982±0.05^e^	2.898±0.21^b^
**H-P**	113.0±10.4^e^	153.3±5.1^d^	288.6±13.0^d^	344.9±13.2^d^	0.327±0.006^c^	0.657±0.022^a^	1.428±0.16^d^	3.321±0.17^a^

SOD: superoxide dismutase; CAT: catalase; GSH: glutathione; AsA: ascorbic acid; H-M: mycelial regeneration; H-V: mycelial pigmentation; H-P: primordium. KA: kojic acid. Data are presented as the means ± SD of three independent experiments. Values with different letters denote a statistically significant difference compared with the control according to multiple comparisons (P < 0.05).

**Fig 6:**
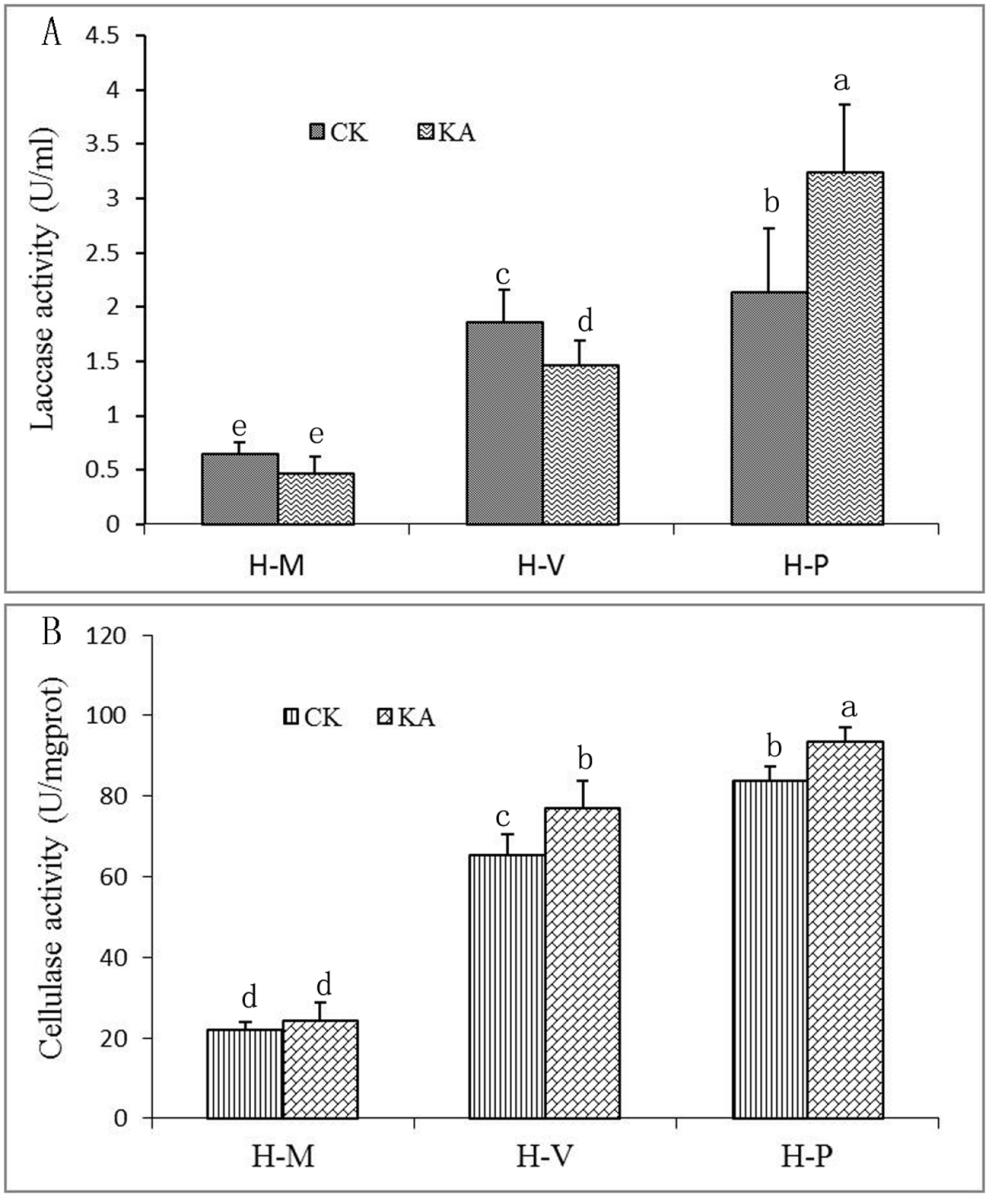
Effects of KA on laccase and cellulase activities at the three developmental stages of *H*. *marmoreus*. After being scraped, damaged mycelia were treated with 15 mL of tap water (CK) or 10 mM KA. Laccase and cellulase activities were detected at the H-M, H-V and H-P stages. The data are presented as the means ± SD of three independent experiments. Bars with different letters denote a statistically significant difference compared with the control according to multiple comparisons (P < 0.05).

The effects of KA on laccase gene expression were analyzed by co-expression analysis [23]. In total, 13 laccase genes were found in the transcriptome of *H*. *marmoreus*. In the H-M transition, no significant differences in expression of most laccase genes due to KA treatment were observed. However, in the H-P transition, expression of most laccase genes was enhanced in the KA group compared to that in the CK group (Table 2). Extracellular laccase activity was also significantly reduced at H-M and H-V but increased by KA at H-P (Fig 6A), which was in accord with the gene expression results. At the same time, we found significantly higher cellulase activity in the KA group than in the CK group at the H-V and H-P transitions, with no significant changes in the H-M transition (Fig 6B).

**Table 2:** Effects of KA on the expression levels of 13 laccase genes at three developmental stages in *H*. *marmoreus*.

Genes No	KA-M/CK-M^a^	KA-V/CK-V^b^	KA-P/CK-P^c^
**GBCL01015765**	0.941	0.760	1.829
**GBCL01019738**	0.741	0.228	0.680
**GBCL01026040**	0.590	0.653	1.120
**GBCL01015764**	0.975	0.567	0.987
**GBCL01011562**	1.178	0.545	1.113
**GBCL01038712**	0.940	0.265	0.881
**GBCL01006585**	0.809	0.348	1.119
**GBCL01002536**	0.802	0.334	1.323
**GBCL01014761**	1.162	0.405	1.304
**GBCL01019987**	1.056	0.861	1.215
**GBCL01019363**	0.373	1.490	2.901
**GBCL01007281**	2.120	0.454	1.271
**GBCL01007282**	1.455	0.461	0.760
**GBCL01002528**	0.535	0.754	0.842

^a^: The ratio of laccase genes at the mycelial knot stage treated with tap water (CK-M) or kojic acid (KA-M);

^b^: the ratio of laccase genes at the mycelial pigmentation stage treated with tap water (CK-V) or kojic acid (KA-V);

^c^: the ratio of laccase genes at the primordium stage treated with tap water (CK-P) or kojic acid (KA-P).

### Signaling pathways involved in mechanical damage

**Fig 7:**
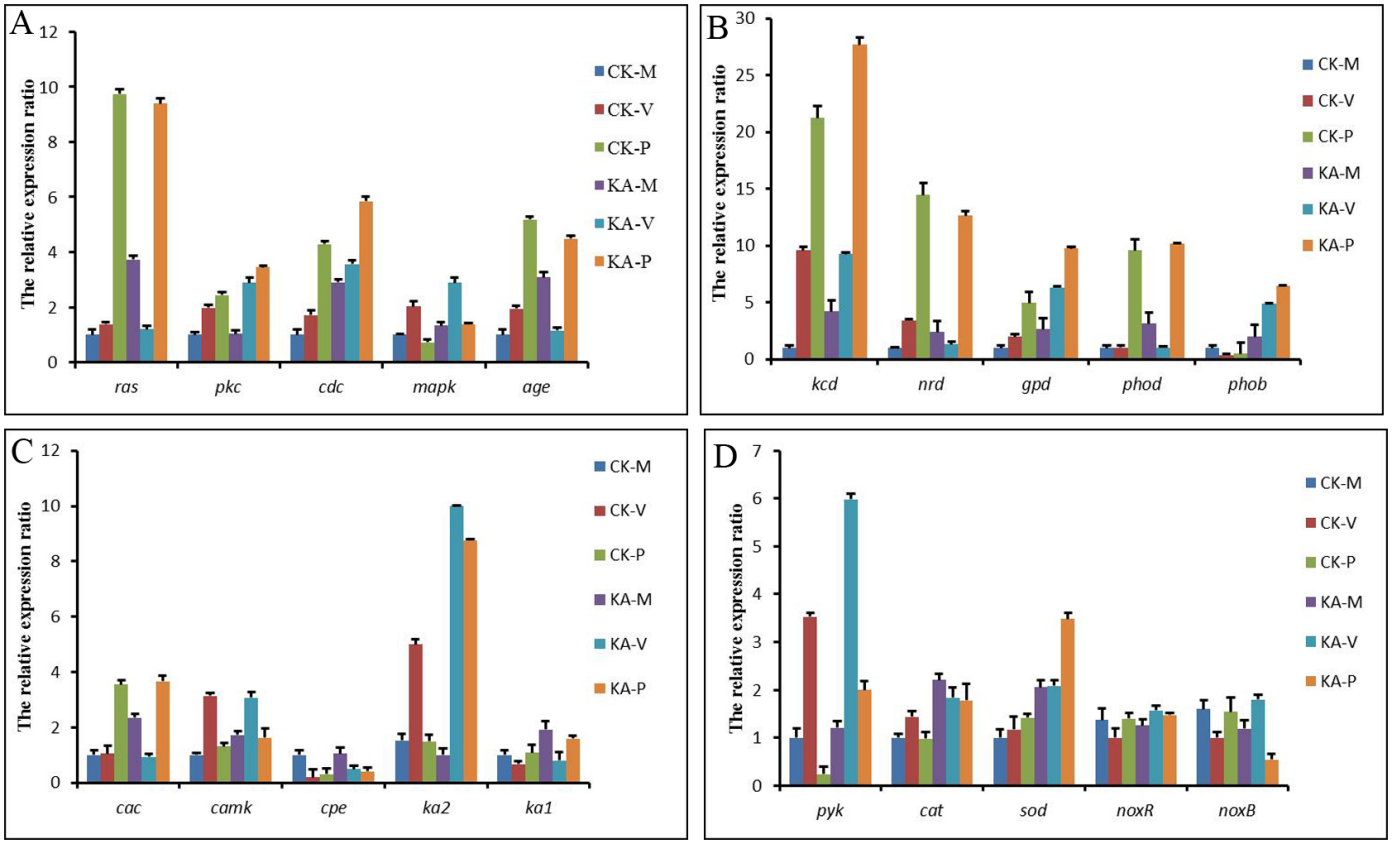
qRT-PCR analysis of gene expression compared to RNA-Seq data for *H*. *marmoreus*. The Y-axis represents the relative expression level in the three stages for the control and KA groups. The unit for the RNA-Seq data is FPKM value. CK-M, CK-V and CK-P: control groups in the three developmental stages of mycelial knot, mycelial pigmentation and primordium, respectively; KA-M, KA-V and KA-P: kojic acid groups in the three developmental stages of mycelial knot, mycelial pigmentation and primordium, respectively. *ras*: Ras protein; pkc: protein kinase C; *cdc*: CDC42 rho GTPase-activating protein; *mapk*: MAPK protein kinase; *age*: RhoA GTPase effector DIA/Diaphanous; *kcd*: alpha-ketoglutarate catabolism dioxygenase; *nrd*: nitrate reductase; *gpd*: glyceraldehyde-3-phosphate dehydrogenase; *phod*: phospholipase D; *phob*: phospholipase B; *cac*: calcium channel; *camk*: CAMK protein kinase; *cpe*: calcium/proton exchanger; *ka1*: Zn(2)-Cys(6) binuclear cluster domain-containing protein; *ka2*: C_2_H_2_-type Zn-finger protein; *pyk*: pyruvate kinase; *cat*: catalase; *sod*: superoxide dismutase; *noxR*: NADPH oxidase regulator; *noxB*: NADPH oxidase B. The error bars represent the standard deviation of three independent replicates.

In this transcriptome database, expression of certain genes involved in several important signaling pathways, including Ca^2+^ signaling (S3 Fig), ROS signaling, MAPK signaling (S4 Fig), and oxylipin signaling, was detected by qRT-PCR (Figs 3 and 7). In fungi, calcium can serve as an important second messenger during growth, development, and cell differentiation. In this study, genes encoding a calcium channel, a CAMK protein kinase, and a calcium/proton exchanger, which are considered key components in calcium signaling in response to different types of stress, were up-regulated in the KA groups (Fig 7).

**Fig 8:**
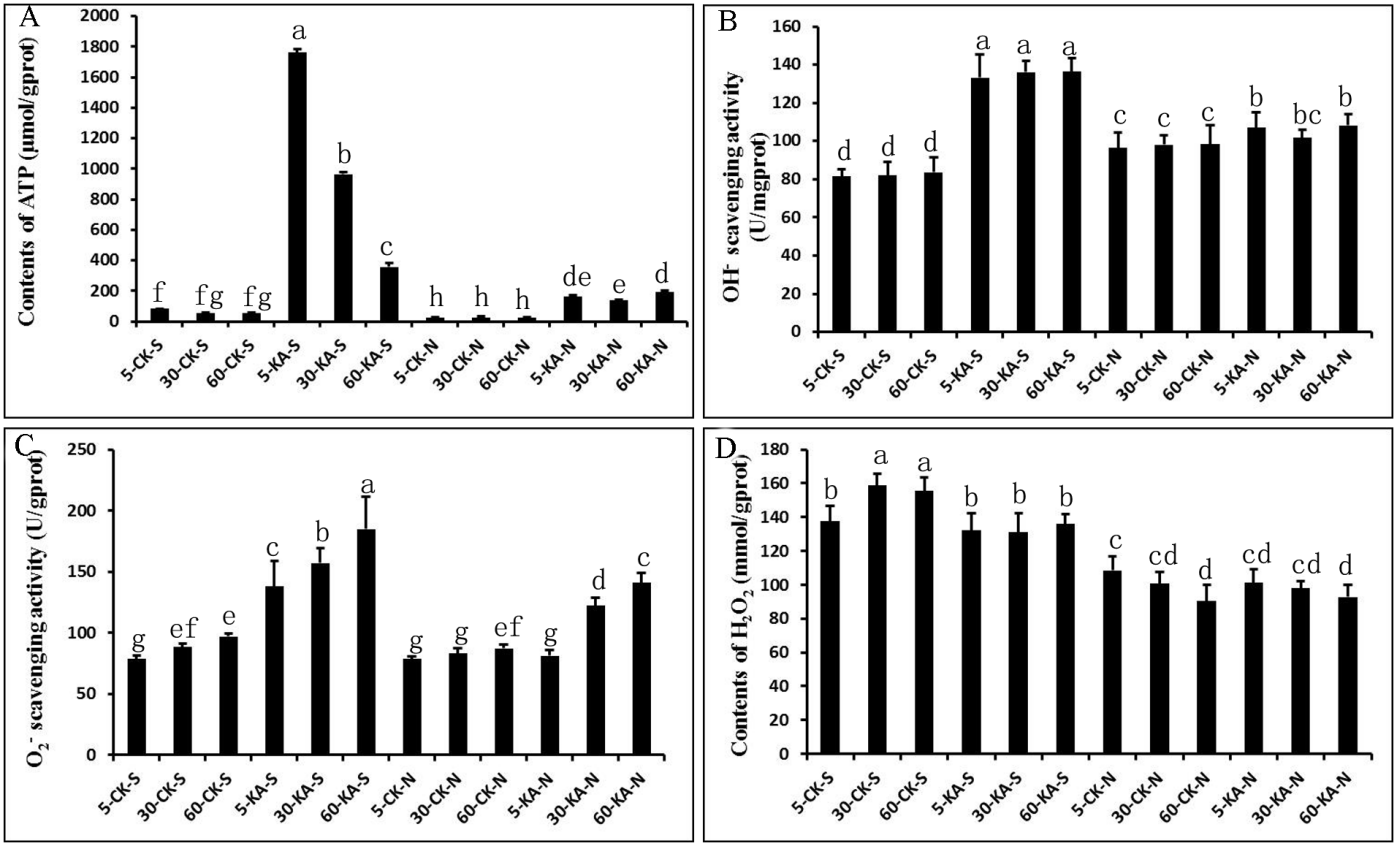
Effects of KA on ATP and ROS contents at the three developmental stages in *H*. *marmoreus*. Mycelia were scraped (S) or unscraped (N), and 15 mL of tap water (W) or KA was added. a: The content of ATP; b: the OH^-^ scavenging activity; c: the O^2-^ scavenging activity; d: the content of H_2_O_2_. The samples were collected at 5 min, 30 min, and 60 min after the different treatments. 5-W-S, 30-W-S, and 60-W-S indicate water added to scraped mycelia at 5, 30, and 60 min; 5-KA-S, 30-KA-S, and 60-KA-S indicate KA added to scraped mycelia at 5, 30, and 60 min; 5-W-N, 30-W-N, and 60-W-N indicate water added to scraped unscraped mycelia at 5, 30, and 60 min; 5-KA-N, 30-KA-N, and 60-KA-N indicate KA added to unscraped mycelia at 5, 30, and 60 min. The data are presented as the means ± SD of three independent experiments. Bars with different letters denote a statistically significant difference compared with the control according to multiple comparisons (P < 0.05).

Genes encoding CMGC/MAPK protein kinase, protein kinase C, CDC42 rho GTPase-activating protein, and Ras protein were also up-regulated by KA (Fig 7). MAPK signaling not only regulates fungal growth and development but is also involved in signal transduction induced by oxidative stress. In fungi, eATP serves as a “stress signal" to activate the MAPK pathway and induce the formation of asexual reproduction structures [7]. We found that the content of eATP was significantly higher in scraped mycelia than in unscraped mycelia, and eATP levels were significantly enhanced by KA treatment after scraping (Fig 8A).

ROS is another important “stress signal” in the mechanical damage response, and ROS production mainly depends on plasma membrane-bound NADPH oxidase (Nox) activity [9–10]. Two genes, encoding an NADPH oxidase regulator NoxR and NADPH oxidase NoxB, were regulated by KA treatment. We also detected -OH and O^2-^ scavenging activity (Fig 8B and 8C) and the content of H_2_O_2_ (Fig 8D). After scraping mycelia, KA significantly reduced the content of H_2_O_2_, and scavenging of -OH and O^2-^ was significantly promoted. Genes encoding alpha-ketoglutarate catabolism dioxygenase, phospholipase B and phospholipase D are all involved in the oxylipin biosynthetic pathway (Fig 7). Lipid metabolism has been proven to play an important role in the response to mechanical damage in plants, animals, and fungi, and we found that genes involved in oxylipin signaling were also up-regulated by KA.

## Discussion

*H*. *marmoreus* is a very important mushroom species, primarily due to its medicinal and organoleptic properties. Stress might be the most important effector for fruiting body initiation in *H*. *marmoreus* [23], and in the *H*. *marmoreus* production process, mechanical damage by scraping of mature mycelia is needed to induce primordium initiation. However, over-injury of mycelia might delay fruiting body development in this species.

In this study, adding KA to scraping-damaged mycelia enhanced the mycelial regeneration ability (Fig 1A) and significantly increased fruiting body production (Fig 1D). KA is an antioxidant produced as a major secondary metabolite by a range of microorganisms [17]. We found that genes encoding SOD and CAT were up-regulated to different levels by KA; the activities of CAT and SOD were increased and the contents of GSH and AsA were significantly higher in the KA than in the CK groups (Table 1). As shown in Fig 3, antioxidant pathways, such as peroxisomes, ascorbate and aldarate metabolism, and glutathione metabolism, were up-regulated in the KA-M and KA-V groups. Furthermore, the content of KA increased significantly after adding KA (Fig 1E), which is in accordance with the results of a previous study [34–35]. Chang et al. [21] found that production of the secondary metabolite KA promoted the response to oxidative stress in fungi. KA was also able to increase cell resistance to oxidative stress and induce repair of damaged tissues [20, 22]. These results suggest that KA might increase antioxidative capacity of injured mycelia and enhance mycelial healing and regeneration in *H*. *marmoreus*.

Oxidative stress caused by mechanical damage may be an important factor inducing fruiting body initiation in some fungi such as *S*. *commune* [2], *S*. *rolfsii* [3] and *G*. *intraradices* [4]. Some reports have demonstrated that damaged cells or tissue can release their constituents or fragments into the extracellular milieu during cellular stress or damage [6, 7, 9, 36]. eATP has been proven to be an important early damage signal in plants, animals and fungi [7, 9, 36], and Medina-Castellanos et al. [7] showed that exposure of *T*. *atroviride* mycelia to eATP induces asexual reproduction structure formation. In our study, we found significantly increased eATP levels in scraped mycelia compared to unscraped mycelia, and adding KA to damaged mycelia further significantly increased the level of eATP (Fig 8A). These results suggest that KA might trigger the damaged mycelium to release more eATP, which is required for the injury response and for inducing asexual developmental in *H*. *marmoreus*.

ROS, which include H_2_O_2_, O^2-^, -OH and singlet oxygen (O_2_) [37–38], play important roles in cell proliferation, signal transduction, cell differentiation, and injury response [39]. However, ROS also affect cellular components via oxidation of proteins, DNA, lipids, and other molecules to induce cell damage or death [11]. In this study, the ROS signaling pathway was down-regulated in the KA groups relative to CK groups. *noxB* was down-regulated by KA, and the concentrations of O^2-^, -OH, and H_2_O_2_ in damaged mycelia were significantly higher in the CK groups than in the KA groups, with significant increases in undamaged KA group mycelia compared to CK group mycelia (Fig 8). These results suggest that KA reduced oxidative harm by decreasing ROS to stimulate damaged mycelial healing and regeneration in *H*. *marmoreus*.

The MAPK and Ca^2+^ signaling pathways also participate in the injury response in fungi [7]. In our study, genes encoding MAPK protein kinase, protein kinase C (PKC), CDC42 rho GTPase-activating protein, Ras protein involved in the MAPK signaling pathway, a calcium channel, CAMK protein kinase, and a calcium/proton exchanger involved in calcium signaling were up-regulated by KA. In filamentous fungi, the MAPK pathway is involved in several processes, such as asexual and sexual reproduction, general stress response, cell fusion, and spore germination [40–41]. In *Magnaporthe grisea*, defective sexual and asexual development results from MAP kinase mutations [42]. Juvvadi et al. [43] determined that PKC is important for the correct localization of the dolipore septum at the septal pore. In basidiomycetes, the septal pore cap, which is a membranous structure located at both sides of the dolipore septum, plays a key role in plug formation after hyphal damage or stress [44]. In addition, calcium plays an important role as a second messenger for wounding and participates in the injury response in *T*. *atroviride* [7], and calcium signaling pathways also have important roles in primordium initiation in *H*. *marmoreus* [23]. These results suggest that KA induces MAPK and calcium pathways to stimulate the transition from mycelium to primordium in *H*. *marmoreus*.

Moreover, lipid metabolism has an important function in the injury responses of plants, animals, and fungi [7, 45–46]. Hernandez-Onate et al. [6] proved that fungi produce oxylipins in response to mechanical damage through a set of enzymes shared with the biosynthetic pathways of plants and animals. In the present study, oxylipin signaling was stimulated by KA treatment, and transcriptional analyses showed that genes encoding phospholipase B, phospholipase D, and α-dioxygenase were up-regulated by KA in *H*. *marmoreus* (Fig 7). Regulation of the oxylipid pathway after mechanical damage is particularly important for plants to initiate defense reactions in a timely fashion [47], and activation of oxylipid signaling might also induce immune responses in plants. In *T*. *atroviride*, genes associated with oxylipin biosynthesis are up-regulated during the injury response, which is similar to activation of the fungal immune response [6]. These results suggest that adding KA to damaged *H*. *marmoreus* mycelia might rapidly activate the defense response after mechanical damage.

In addition, genes involved in carbon metabolism, such as pyruvate kinase and glyceraldehyde 3-phosphate dehydrogenase, were activated by KA (Fig 7). Laccase activity was significantly increased at the H-P stage by KA, and cellulase activity was significantly increased at the H-V and H-P stages by KA (Fig 6). Laccase and cellulase play important roles in lignin and cellulose degradation in fungi [48–49] and also in mycelial maturation and fruit-body growth in *H*. *marmoreus* [49–51]. These results suggest that KA might activate primary metabolism to increase fruiting body production in *H*. *marmoreus*.

## Conclusion

**Fig 9:**
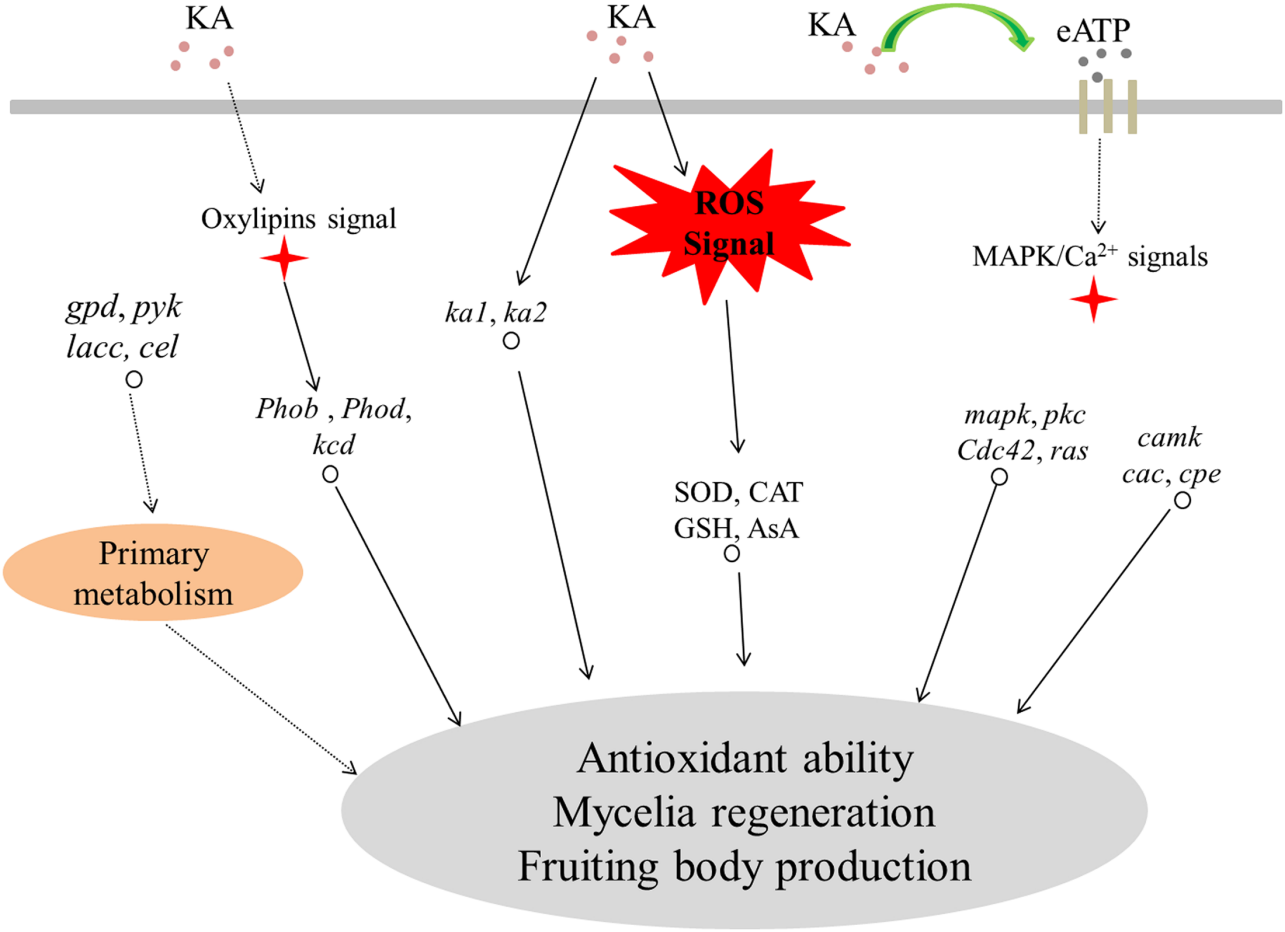
Schematic representation of the injury response in *H*. *marmoreus* after kojic acid treatment. Adding KA causes mycelia to respond to injury faster by increasing the content of eATP (Fig 8). The mycelial regeneration capacity is enhanced by increasing antioxidative enzyme activity (SOD and CAT) and antioxidants (GSH and AsA) (Table 1) as well as the KA content (Fig 1). KA also activates signaling pathways including the MAPK pathway (*mapk*), Ca^2+^ signaling pathway (*cam*, *cac*, *cpe*), and oxylipin signaling pathway (*phoa*, *phob*, *phoc* and *phod*) (Figs 5 and 7). In addition, *pyk*, *gpd*, *lacc*, and *cel* were up-regulated by KA (Figs 6 and 7). The solid line indicates that the results were confirmed by transcriptomic analysis and experimental data; the dotted line indicates that the results were only confirmed by transcriptomic analysis.

Mechanical damage can stimulate fruiting body initiation in fungi. However, the molecular mechanism invoked in response to mechanical damage is less well understood. In this work, we found that adding KA to damaged mycelia induced mycelial regeneration. A schematic model by which KA regulates the fruiting body of *H*. *marmoreus* is shown in Fig 9. It was found that adding KA increased eATP levels to stimulate damaged mycelia to quickly respond to the injury, acting as a “stress signal”. The mycelial regeneration capacity was enhanced by increasing SOD and CAT activities and the contents of GSH and AsA. KA also increased the content of KA and resistance to oxidative injury in *H*. *marmoreus*. Genes involved in MAPK (*mapk*, *cdc42*, *ras*, *pkc*), Ca^2+^ (*camk*, *cac* and *cpe*) and oxylipin (*phob*, *phod* and *kcd*) signaling were activated by KA, and these pathways might play important roles in fruiting body initiation in *H*. *marmoreus*. In addition, expression of *pyk*, *gpd*, *cel* and *lacc* was affected by KA treatment at different developmental stages, suggesting that KA regulates primary metabolism for fruiting body production.

## Supporting information

S1 Fig Gene Ontology classification of the *H*. *marmoreus* transcriptome. (TIF)

S2 Fig COG functional categories of *H*. *marmoreus* unigenes. (TIF)

S3 Fig Calcium signaling pathway identified by KEGG annotation (http://www.kegg.jp/kegg-bin/show_pathway?ko04020). The red boxes indicate that the genes identified in the transcriptome of *H*. *marmoreus* are annotated in the metabolic pathways.
(TIF)

S4 Fig MAPK signaling pathway identified by KEGG annotation (http://www.kegg.jp/kegg-bin/show_pathway?ko04011). The red boxes indicate that the genes identified in the transcriptome of *H*. *marmoreus* are annotated in the metabolic pathways.
(TIF)

S1 Dataset All unigenes from the *H*. *marmoreus* transcrptome. (XLSX)

S2 Dataset The KEGG annotations in the six samples of *H*. *marmoreus*. (XLSX)

S3 Dataset The DEGs information in the 10 subclusters of *H*. *marmoreus*. (XLS)

S1 Table Primers used for quantitative real-time PCR. (DOCX)

S2 Table The functional annotation for 27 unigenes from six DGE libraries. (DOCX)
