# Supplementary figures and images for: Kojic acid-mediated damage responses induce mycelial regeneration in the basidiomycete Hypsizygus marmoreus
Source: PLoS One. 2017 Nov 8;12(11):e0187351. doi: 10.1371/journal.pone.0187351 (PMC5678884; doi:10.1371/journal.pone.0187351)

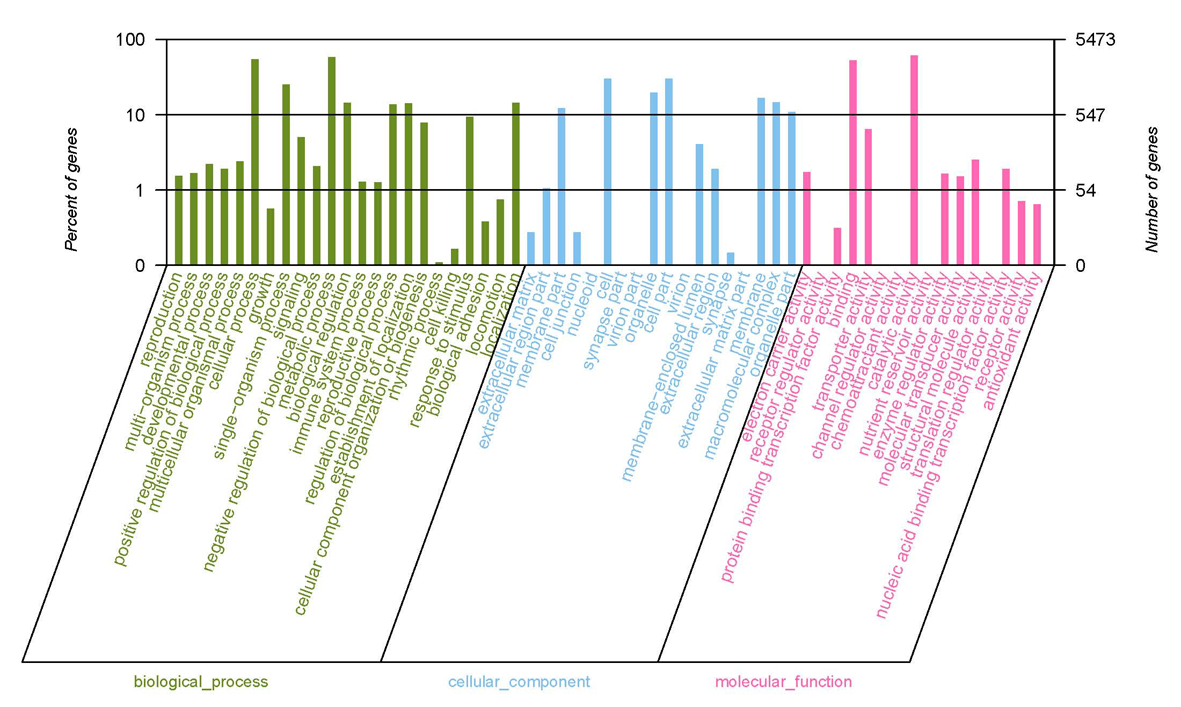

Supplement: S1 Fig — (TIF) [file pone.0187351.s001.tif]

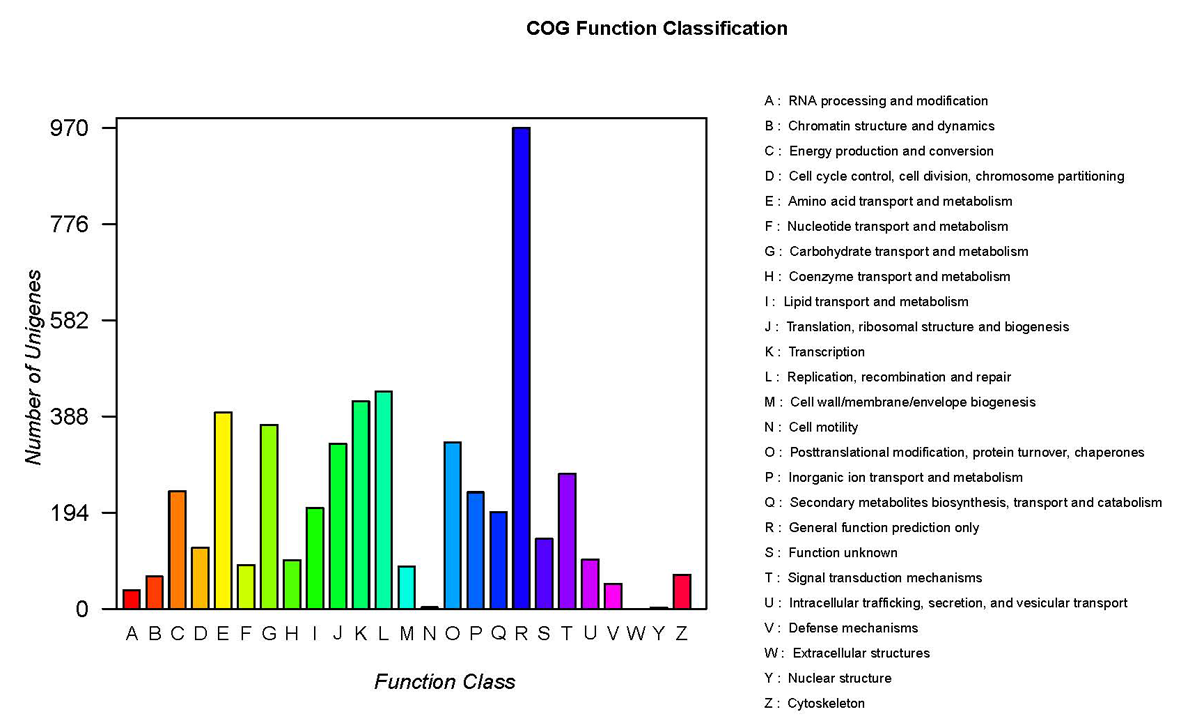

Supplement: S2 Fig — (TIF) [file pone.0187351.s002.tif]

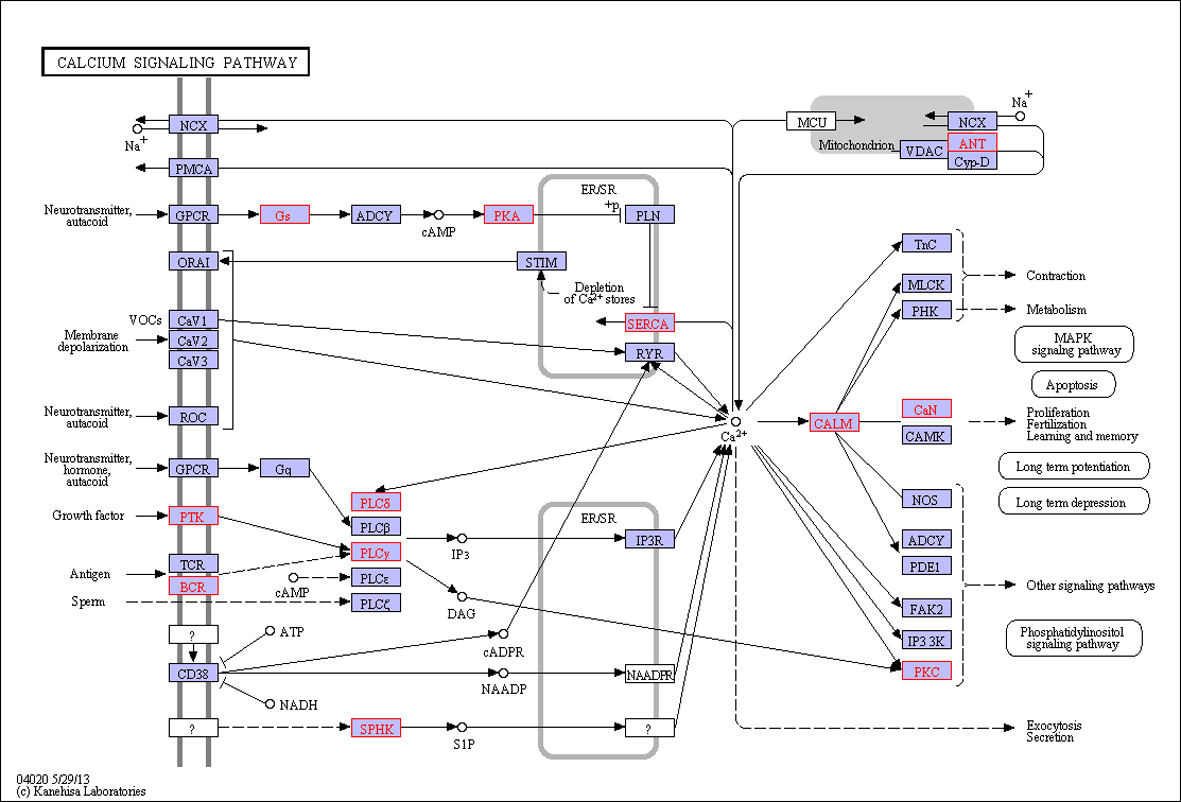

Supplement: S3 Fig — The red boxes indicate that the genes identified in the transcriptome of H. marmoreus are annotated in the metabolic pathways. (TIF) [file pone.0187351.s003.tif]

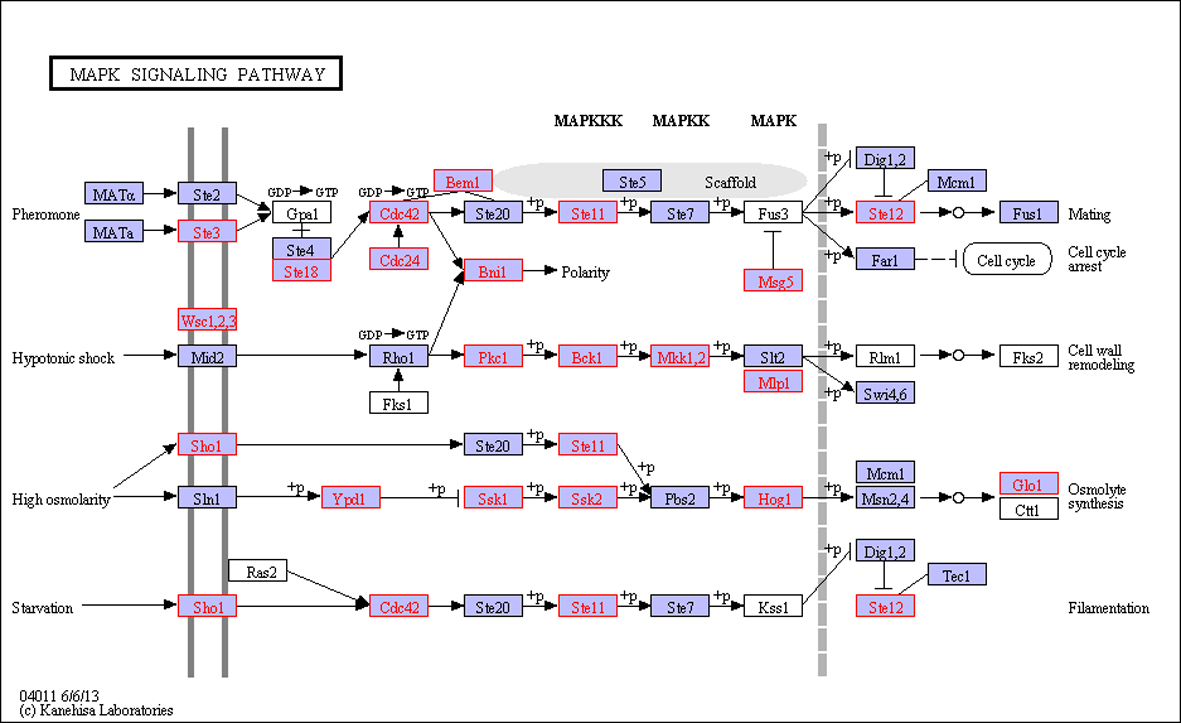

Supplement: S4 Fig — The red boxes indicate that the genes identified in the transcriptome of H. marmoreus are annotated in the metabolic pathways. (TIF) [file pone.0187351.s004.tif]
