# Supplementary material for: Kojic acid-mediated damage responses induce mycelial regeneration in the basidiomycete Hypsizygus marmoreus
Source: PLoS One. 2017 Nov 8;12(11):e0187351. doi: 10.1371/journal.pone.0187351 (PMC5678884; doi:10.1371/journal.pone.0187351)
Supplement: S1 Table — (DOCX) [file pone.0187351.s008.docx]

**S1 Table. Primers used for quantitative real-time PCR.**

| Unigene name | Sequence (5^’^-3^’^) | Accession No. |
| --- | --- | --- |
| comp14816_c1 | CCAAATACCCAATACGAACAG  AACCATCGAGACCTCCACTT | GBCL01017519 |
| comp16006_c0 | TAAATGATTTGGTGGAGGC  CATCGGTGAAACTGAGGC | GBCL01014403 |
| comp15783_c0 | AGGACAAACAAGGATGGAGGAC  TATGGCAGACCGAAGACGAG | GBCL01028590 |
| comp17060_c2 | ACTTGAAGCCTGCCAACCTC  TCCTTGCCTCCTGGAACACT | GBCL01019135 |
| comp17213_c0 | CCCGAGATTGTGGCATTTGT  TTGCCTTTGCTCCAGGGTTC | GBCL01000803 |
| comp16518_c0 | ACCTGCATCAGGCGGTACTACGA  CTGGACCAATGGGCATCACG | GBCL01030806 |
| comp18046_c0 | ACCGCCTCCTCAAGACAACA  CCACGCCTAAGCCAACTCCC | GBCL01008459 |
| comp17081_c0 | TTCATCGGACTCTGAGGTGG  CGAGATAATCGGCTGTAACGT | GBCL01010171 |
| comp98391_c0 | TCCAACCTTCTTCGGATGTG  ATACGTGCTTGCGGGTGTAG | GBCL01006089 |
| comp15221_c0 | CATCGCTGCGGTATCTTTAT  CGACCCAATAGTTGTTGAGAAT | GBCL01038488 |
| comp14352_c0 | CAACCGTCCGTTTCTCCACT  CCAATCCCAATTACCTTCCTC | GBCL01039326 |
| comp158105_c0 | GTCACGCAAACCATACATTC  CACAACTAACCAAGCCCAAC | GBCL01008983 |
| comp15932_c0 | AGCAAGCCTTCCGACGAGCAA  CGACGGCGATGTAGCCAGCA | GBCL01010302 |
| comp16003_c0 | AGAAATGCCAACCCAAATCC  AGTCTCCGCTCGCATAACCC | GBCL01012047 |
| comp16378_c0 | TGTCCCGAGGTCTCCTATTT  GTTCCGATAACCTTTCCACC | GBCL01013141 |
| comp9246_c0 | GCGTCCAGGGCTAGAAGAAG  ACAAGGTGTCTCCGCCAAAT | GBCL01011994 |
| comp16883_c0 | GTTTGTGGTGAATGGCGAGGTT  GCTTGTGCCGTGATCGAGTG | GBCL01030867 |
| comp15724_c0 | GCTACGCTCCTCGACGCTGAT  CATTGTTCGCACCGTGGCTC | GBCL01029063 |
| comp18252_c0 | CCAGGACGCCACAACAGAAC  CGACTCCACCACATCCAAGTA | GBCL01022126 |
| comp15921_c0 | CATCCCATGAGCCAGTTTAGTT  CCCTGTCCGAAGACATTACG | GBCL01028587 |
| 18s ribosomal protein | GAGGGACCTGAGAAACG  ATAAGACCCGAAAGAGCC | KC510993 |
