# Supplementary material for: Kojic acid-mediated damage responses induce mycelial regeneration in the basidiomycete Hypsizygus marmoreus
Source: PLoS One. 2017 Nov 8;12(11):e0187351. doi: 10.1371/journal.pone.0187351 (PMC5678884; doi:10.1371/journal.pone.0187351)
Supplement: S2 Table — (DOCX) [file pone.0187351.s009.docx]

**S2 Table. The functional annotation for 27 unigenes from six DGE libraries.**

| EST ID | Gene function | E-value | Accession no. | Organism |
| --- | --- | --- | --- | --- |
| comp18665_c2 | phospholipase B | 0 | XP_002911698 | *Coprinopsis cinerea* |
| comp18259_c0 | phospholipase D | 0 | EIW80924 | *Coniophora puteana* |
| comp17808_c0 | phospholipase A-2-activating protein | 0 | XP_001833148 | *Coprinopsis cinerea* |
| comp16330_c0 | phosphoinositide-specific phospholipase C | 0 | XP_001840483 | *Coprinopsis cinerea* |
| comp16003_c0 | NADPH oxidase regulator NoxR | 0 | XP_001877437 | *Laccaria bicolor* |
| comp16378_c0 | NADPH oxidase B | 0 | EKM56649 | *Laccaria bicolor* |
| comp15932_c0 | glutathione reductase | 0 | XP_001878186 | *Laccaria bicolor* |
| comp130666_c0 | glutathione peroxidase | 0 | CAA27558 | *Mus musculus* |
| comp14816_c1 | L-ascorbate oxidase | 0 | XP_001832966 | *Coprinopsis cinerea* |
| comp15724_c0 | cytochrome c peroxidase | 0 | XP_001877525 | *Trametes versicolor* |
| comp15783_c0 | calcium channel | 0 | XP_001830774 | *Coprinopsis cinerea* |
| comp18046_c0 | CAMK/CAMKL/GIN4 protein kinase | 0 | XP_001832241 | *Coprinopsis cinerea* |
| comp17081_c0 | calcium/proton exchanger | 0 | EJD07523 | *Fomitiporia mediterranea* |
| comp18252_c0 | Ras protein | 0 | BAD04019 | *Schizophyllum commune* |
| comp17060_c2 | CMGC/MAPK protein kinase | 0 | XP_001828839 | *Coprinopsis cinerea* |
| comp16006_c0 | protein kinase C | 0 | XP_001876242 | *Laccaria bicolor* |
| comp14519_c0 | GTPase | 0 | XP_001837733 | *Coprinopsis cinerea* |
| comp16518_c0 | CDC42 rho GTPase-activating protein | 0 | XP_001876797 | *Laccaria bicolor* |
| comp17213_c0 | RhoA GTPase effector DIA/Diaphanous | 0 | XP_001877017 | *Laccaria bicolor* |
| comp14352_c0 | catalase | 0 | XP_001889495 | *Laccaria bicolor* |
| comp158105_c0 | superoxide dismutase | 0 | WP_002355345 | *Enterococcus faecalis* |
| comp15221_c0 | Zn(2)-Cys(6) binuclear cluster domain-containing protein | 0 | AEN14436 | *Lentinula edodes* |
| comp14401_c0 | C2H2-type Zn-finger protein | 0 | ABF18162 | *Aedes aegypti* |
| comp9246_c0 | alpha-ketoglutarate catabolism dioxygenase | 0 | AFR96611 | *Cryptococcus neoformans* |
| comp16883_c0 | nitrate reductase | 0 | XP_001840500 | *Coprinopsis cinerea* |
| comp15921_c0 | citrate synthase | 0 | XP_001880864 | *Laccaria bicolo* |
| comp98391_c0 | pyruvate kinase | 0 | XP_753115 | *Enterococcus faecalis* |
